# Coronavirus-19 and malaria: The great mimics

**DOI:** 10.4102/phcfm.v12i1.2501

**Published:** 2020-08-05

**Authors:** Tapiwanashe Kusotera, Trust G. Nhengu

**Affiliations:** 1 Department of Medicine, College of Health Sciences, University of Zimbabwe, Harare, Zimbabwe; 2 Musiso Hospital, Masvingo Province, Zimbabwe

**Keywords:** COVID-19, Coronavirus, malaria, SARS-CoV-2 RDT kits, screening

## Abstract

The use of SARS-CoV-2 rapid diagnostic test (RDT) kits by some African countries for screening has raised serious concerns over their role in malaria areas. Coupled with a lack of adequate personal protective equipment and the scarcity of knowledge on the possible interaction between malaria and COVID-19 both in terms of presentations and shared symptoms, this has left many frontline health workers with fears and anxieties. Several anecdotal reports have already raised questions pertaining to possible false-positive COVID-19 results in proven malaria cases by use of SARS-CoV-2 RDT kits with huge costs to already constrained budgets. The report raises concerns on the use of SARS-CoV-2 kits in malaria areas in terms of cost, to prompt research, allay fears and guide policy during this pandemic and beyond.

## Introduction

The Coronavirus disease 2019 pandemic has been reported in over 200 countries. In Africa, over 100 000 cases have been reported and the number continues to rise.^1^ Unique to Africa is the widespread distribution of malaria with isolated seasonal outbreaks. The World Health Organization (WHO) has warned that up to 769 000 people could die of malaria this year in sub-Saharan Africa, more than double the number of deaths in 2018, if the focus on COVID-19 leads to a disruption of interventions for malaria.^2^ With the advent of SARS-CoV-2 virus and the difficulties associated with diagnosis in terms of resources, trained personnel and sampling errors, there has been a huge demand for quicker and easier point-of-care testing to direct patient care. WHO does not recommend the use of SARS-CoV-2 rapid diagnostic tests (RDT) to guide patient care owing to low sensitivity and specificity.^3^ Some low- to medium-income countries have resorted to using RDT kits for screening and diagnosis because of the shortage of rapid test polymerisation chain reaction kits (RT-PCR kits).

COVID-19 is an emerging disease in Zimbabwe. A limitation to identifying cases has been the lack of RT-PCR tests to map the outbreak. Until recently, testing has been limited to people returning from travel abroad, who were the original cases, and contacts were traced from these cases. To date (30 May 2020), 44 635 tests have been carried out, and 174 people confirmed to be positive by RT-PCR with 4 deaths.^4^ Positive cases were mainly residents of Harare (the capital city), Bulawayo (second biggest city), Victoria Falls (major tourist destination) and Masvingo (transit town from South Africa). Over 100 cases have been identified from several quarantine centres in the last 4 days and most of these were travellers from South Africa and Britain. Recently acquired antibody tests have been sent to district hospitals for use with suspected cases that have typical symptoms of fever, cough and shortness of breath. Patients presenting with fever may be suspected of having COVID-19 when they are more likely to have malaria, which is more common. Table 1 shows shared symptoms for both malaria and COVID-19 disease, while Table 2 shows the case definition for COVID-19 disease.

**TABLE 1:** Malaria and COVID-19 shared symptoms.

Disease	Symptoms
Malaria	• Fever, headache, tiredness respiratory distress, aches and pains
COVID-19	• Fever, headache, tiredness, aches and pains, respiratory distress

*Source:* World Health Organisation^5,6^

**TABLE 2:** The current case definitions for COVID-19 disease in Zimbabwe.

Cases	Definitions
1. Suspected cases meet one of the following criteria	A person with acute respiratory illness AND a history of travel or residence in a location reporting community transmission of COVID-19 disease during the 14 days prior to symptom onset. A patient with any acute respiratory illness AND having been in contact with a confirmed/probable COVID-19 case in the last 14 days prior to symptom onset. A patient with a severe acute respiratory illness AND in the absence of an alternative diagnosis that fully explains the clinical presentation.
2. Confirmed case	A person with laboratory confirmation of COVID-19 infection, irrespective of clinical signs and symptoms.

*Source*: Ministry of Health and Child Care^7^

This report calls to attention the dangers of using SARS-CoV-2 RDT kits in areas of high malaria burden and seeks to prompt research as well as direct policy and guidelines going forward.

## SARS-CoV-2 testing in malaria areas

Malaria is a major public health problem in Zimbabwe, accounting for 1594 deaths between 2012 and 2015.^8^ Transmission in the country differs from year-round to epidemic-prone outbreaks depending on the region. In 2019, there were 117 715 malaria and 127 deaths compared to current figures in 2020 of 170 303 and 152 deaths, a 44.7% increase. In addition, 201 outbreaks have been recorded throughout the country mostly from malarious areas such as Manicaland, Masvingo and Mashonaland East.^8^

Malaria cases declined in Zimbabwe from 155 per 1000 people to 22 per 1000 between 2003 and 2013 because of strong government funding.^8^ As the country continues to face serious economic challenges and deterioration of health delivery systems, malaria outbreaks have steadily increased. The diagnosis of malaria in Zimbabwe is mainly done using RDT kits. Two types are currently being used: PARACHECK (Paracheck-Pf^®^ Orchid Biomedical Systems, Goa, India) and SD BIOLINE (Standard Diagnostics, Inc.), with the former being preferred by the Ministry of Health and Child Care.^8^ The sensitivities for both tests approach 99% (97.7% – 100%) at parasitemia of above 1000/micromoles.^10^ Malaria Giemsa stain microscopy is regarded the gold standard for diagnosis. Difficulties in the technical skill required to detect malaria parasites on microscopy have led to most district hospitals relying heavily on the use of the RDT kits.

The limited SARS-CoV-2 testing capability in the country at present, and its centralisation to the National Laboratory, means that district hospitals in Zimbabwe will have to rely on the rapid SARS-CoV-2 test for screening patients as well as their frontline staff. Two types of SARS-CoV-2 RDT kits are currently available: antigen and antibody detector kits. The antigen test identifies viral proteins expressed by the corona virion from a sample taken from a patient’s respiratory tract. The sensitivity of these tests depends on the quality of sample taken, virus titre and the stage of the infection. Their overall sensitivity as reported by the WHO is between 34% and 80%.^3^

The antibody RDT kits work by identifying the antibodies to the virus. Their efficacy depends on the immune response mounted by the host. In certain disease states like HIV and malnutrition where the patient may mount a poor antibody response, there could be false-negative results. The Zimbabwe National Microbiology Reference Laboratory confirmed that four types of COVID-19 rapid test kits have been evaluated for rapid antibody detection in whole blood and successfully passed the validation procedure.^9^

### Ethical consideration

This article followed all ethical standards for a research without direct contact with human or animal subjects

## Discussion

People have presented at district hospitals with fever and other symptoms such as shortness of breath and general body weakness. Sometimes, there is a history of travel from South Africa. When South Africa started its lockdown, thousands of people decided to return home to ride out the lockdown. An estimated 13 000 people returned to Zimbabwe, most of whom did not self-isolate on return. In two cases known to the authors, the malaria RDT was positive, malaria slides were positive and antibody tests for COVID-19 were positive. There have been stories in the media of other areas where communities were panicking because of suspicion of pneumonia related to COVID-19, which turned out to be malaria, positive on slide and RDT. News reports confirmed that the provincial health services:

… refuted claims suggesting that there are 6 people who died of coronavirus in the province this year saying it was malaria. … We have noted an upward trend in malaria cases that we have recorded this year as they are overshooting three-fold when compared to last year’s … from February up to end of March, we had recorded six malaria-related deaths … [*and*] a total of 896 cases throughout the province … It is possible for malaria to be mistaken for coronavirus considering that there are some malaria symptoms which can also be exhibited by people who would have contracted coronavirus. (*Pindula News*, 10 April 2020)^11^

The authors were unable to locate any literature reporting cross-reactivity between malaria and COVID-19 disease on rapid test kits.

The implications of false-positive SARS-CoV-2 are costly. The need to further screen the patient’s family and trace all contacts delays in the turn-around time for the results, managing the fear and anxieties of the patient and family. The patients would have to be treated as COVID-19 until a confirmatory test result is reported. The diversion of scarce resources and pressure on constrained budgets can be overwhelming. For health workers, the lack of adequate personal protective equipment (PPE) for frontline health workers attending such patients has its own anxieties leading to compromised healthcare. On the contrary, all the other conditions that patients present with, such as malaria and other treatable diseases, become neglected or mistreated. Health workers would have to self-isolate for days further compromising staffing at health facilities. In some cases, patients would have to be discharged before complete recovery owing to lack of adequate PPE to continue attending to them.

One of the features of the management of the current pandemic has been the readiness of the research community to undertake studies and publish so that they are incorporated into patient management and policy immediately. Clinicians and scientists have shared their clinical experience in webinars, podcasts and on social media sites. Ministries of Health and the policy community have shared medical information with the public to an extent that has not happened before. In our context, without resources to undertake research in the usual way, and the unique context of COVID-19 in Africa compared to Europe, North America or China, we should be exploring new ways, such as crowd-sourcing data, to understand and manage our experiences.

The context in which patient care is taking place during the pandemic is changing constantly. Health systems in Africa could potentially be overwhelmed by COVID-19, yet we are already facing the consequences of other disease outbreaks. In view of this, we recommend that studies be conducted in malaria areas on innovative ways to educate health personnel and the community; manage distress brought about by fear of contracting COVID-19 and potentially losing one’s life; and develop resources for evidence-based guidelines and policy regarding the use of SARS-CoV-2 kits in high malaria areas, especially in a context of constant change. What we put in place now will assist us in managing the next pandemic.
